# Lateral to End-on Conversion of Chromosome-Microtubule Attachment Requires Kinesins CENP-E and MCAK

**DOI:** 10.1016/j.cub.2013.06.040

**Published:** 2013-08-19

**Authors:** Roshan L. Shrestha, Viji M. Draviam

**Affiliations:** 1 Department of Genetics, University of Cambridge, Cambridge, CB2 3EH, UK

## Abstract

**Background:**

Proper attachment of chromosomes to microtubules is crucial for the accurate segregation of chromosomes. Human chromosomes attach initially to lateral walls of microtubules. Subsequently, attachments to lateral walls disappear and attachments to microtubule ends (end-on attachments) predominate. While it is known in yeasts that lateral to end-on conversion of attachments occurs through a multistep process, equivalent conversion steps in humans remain unknown.

**Results:**

By developing a high-resolution imaging assay to visualize intermediary steps of the lateral to end-on conversion process, we show that the mechanisms that bring a laterally bound chromosome and its microtubule end closer to each other are indispensable for proper end-on attachment because laterally attached chromosomes seldom detach. We show that end-on conversion requires (1) the plus-end-directed motor CENP-E to tether the lateral kinetochore onto microtubule walls and (2) the microtubule depolymerizer MCAK to release laterally attached microtubules after a partial end-on attachment is formed.

**Conclusions:**

By uncovering a CENP-E mediated wall-tethering event and a MCAK-mediated wall-removing event, we establish that human chromosome-microtubule attachment is achieved through a set of deterministic sequential events rather than stochastic direct capture of microtubule ends.

## Highlights

• Lateral kinetochores seldom detach and are gradually converted to end-on kinetochores
• CENP-E is essential for tethering of kinetochores to walls but not microtubule ends
• MCAK is required for removing laterally attached microtubules

## Introduction

Kinetochores are large multiprotein structures that act as platforms for microtubules to attach and power chromosome movement. A mature vertebrate kinetochore is bound to the ends of ∼20–30 microtubules, in an end-on fashion [1, 2]. However, vast majority of kinetochores attach to lateral walls and not the ends of microtubules [3, 4]. The steps through which a laterally attached human kinetochore is ultimately converted into an end-on-attached one have remained elusive. When a lateral kinetochore undergoes poleward movement [3, 5], the kinetochore is brought to a microtubule-rich area near the pole where direct end-on capture can occur. However, such poleward movement in itself is unlikely to be sufficient for establishing end-on attachments in all kinetochores because kinetochores arrange around the spindle [4, 6], and not all kinetochores make poleward excursions.

Tethering of kinetochores to microtubule ends requires the loop region of the outer kinetochore protein HEC1/Ndc80 (HEC1^Ndc80^) [7–12]. Human kinetochores bound to lateral walls were reported in cells lacking CENP-E, MCAK, Bub1, and SKAP/astrin [13–16]. But how a human kinetochore bound to the walls of microtubules becomes tethered to the ends of microtubules has not been reported so far. Because multiple microtubule ends attach to a human kinetochore, it is likely that end-on conversion is a multistep and gradual process wherein a kinetochore progressively loses contact with microtubule walls as it simultaneously gains interaction with microtubule ends. In yeasts, where a single microtubule engages with a kinetochore, lateral to end-on conversion is recognized as a multistep process that was visualized with a live-cell assay [17, 18]. However, a live-cell assay to study the temporal evolution of human kinetochore-microtubule interaction has not been described so far.

We establish a live-cell-based methodology to study the lateral to end-on conversion of chromosome-microtubule attachments (hereafter termed end-on conversion). We show that end-on conversion is a gradual process during which lateral kinetochores seldom detach. By analyzing the fate of lateral kinetochores in CENP-E- or MCAK-depleted cells, we show evidence for two distinct events in the end-on conversion process. MCAK is required for a wall-removal event because lateral kinetochores of MCAK-depleted cells can achieve partial end-on status but cannot fully remove contact with microtubule walls. In contrast, CENP-E is required for a wall-tethering event as lateral kinetochores of CENP-E-depleted cells undergo premature detachment from microtubule walls but not ends. We propose a molecular model in humans for the end-on conversion process, wherein CENP-E and MCAK are required for distinct steps of the process.

## Results

### Lateral Kinetochores Are Gradually Converted into End-on Kinetochores

**Figure 1:**
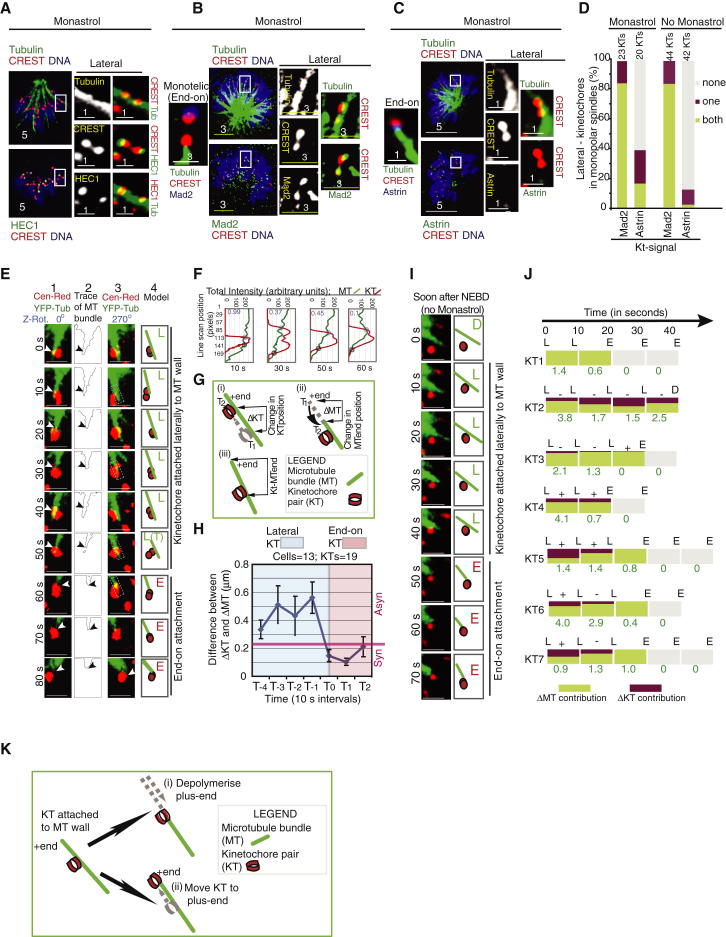
Lateral Kinetochores Are Gradually Converted into End-on Kinetochores (A–C) Fluorescence micrographs showing lateral kinetochores on microtubule walls (A) and levels of Mad2 (B) and astrin (C) on lateral kinetochores in monastrol-treated HeLa cells immunostained with antibodies as indicated and stained with DAPI for DNA. Scale bar measurements are indicated. Insets show 3× magnifications. (D) Graph of percentage of lateral kinetochore pairs with both, one, or neither (none) of the pair displaying Mad2 or astrin in monopolar spindles of monastrol-treated or untreated cells as indicated. (E) Deconvolved single-plane images of a Z stack time-lapse movie of a lateral kinetochore (red) and associated microtubule bundle (green) (column 1) in monastrol-treated HeLa^YFP-Tub; Cen-Red^ cells. Trace of tubulin signal (column 2) illustrates shrinkage and growth of the microtubule bundle. Note the synchronous movement of kinetochore and MT end after end-on attachment (arrows mark kinetochore-attachment site). Images in column 3 are 270° Z-rotated view of column 1. Models in column 4 mark the lateral (L) or end-on (E) kinetochore being tracked. Scale bars represent 2 μm. (F) Graph of total intensity of tubulin and CENP-B signals along the length of rectangular segment (dashed white) (see column 3 of E). Values in purple indicate ratio of tubulin intensities measured at positions before (blue circle) and after (gray circle) the site of kinetochore interaction with microtubule. (G) Illustration of changes in positions of kinetochore (ΔKT) or microtubule-end (ΔMT) between consecutive time frames (T_1_ and T_2_) and distance between the kinetochore attachment site and the microtubule plus end (KT-MT end). (H) Graph of difference between ΔKT and ΔMT through time during end-on conversion of a lateral kinetochore. T_0_ indicates first time point of end-on attachment. Asynchronous (asyn) and synchronous (syn) behaviors indicate greater and lesser than 0.25 μm, respectively, of ΔKT-ΔMT absolute values. (I) Deconvolved single-plane images of a Z stack time-lapse movie of a lateral kinetochore (red) and the associated microtubule bundle (green) (left panels) in monopolar spindles of HeLa^YFP-Tub; Cen-Red^ cells soon after nuclear envelope breakdown (NEBD). Models (right panels) mark lateral (L) or end-on (E) kinetochore being tracked. (J) Temporal evolution of end-on conversion in seven lateral kinetochores selected at random. Fractional contribution of ΔMT (height of green box) and ΔKT (height of red box) to change in KT-MT end distance in μm (green) is shown. + and – indicate direction of kinetochore movement toward plus and minus end of the MT, respectively. (K) Model showing two distinct events, microtubule shrinkage and kinetochore gliding, for bringing the lateral kinetochore and the microtubule end closer to each other. Error bars represent the SEM across cells. See also Table S1 and Figure S1.

To develop a live-cell assay for studying end-on conversion in human cells, we first investigated the state of kinetochore-microtubule (KT-MT) attachments in monopolar spindles of monastrol-treated cells using fixed-cell assays. Monopolar spindles cannot form bioriented KT-MT attachments, and they present kinetochores in a floret-like configuration that allows easy analysis of individual KT-MT attachments. To study KT-MT attachment status, we immunostained cells using antibodies against HEC1^Ndc80^ (an outer-kinetochore marker), β-tubulin, and CREST antisera. In monastrol-treated monopolar spindles, ∼25% of kinetochore pairs (n_cells_ = 18) were bound to microtubule walls, as ascertained by the signal overlap between tubulin and HEC1^Ndc80^ along the outer-kinetochore surface and also by the extension of tubulin signal beyond the site of kinetochore interaction (Figure 1A). We term these as “lateral kinetochores,” wherein at least some, if not all, of the microtubule ends extended beyond the kinetochore attachment site by >0.3 μm. In contrast, at “end-on kinetochores,” tubulin signal terminated at the first site of contact with the kinetochore signal (Figures 1B and 1C). To ascertain that detached kinetochores that nonspecifically overlap with microtubules are not wrongly scored as lateral kinetochores, we analyzed KT-MT attachments in conditions that detach kinetochores from microtubules. For this purpose, we depleted two core kinetochore proteins, Mis12 and HEC1^Ndc80^, that are essential for KT-MT attachment [19, 20]. As expected, a 4-fold increase in detached kinetochores was observed after the depletion of Mis12 or HEC1^Ndc80^ or codepletion of Mis12 and HEC1^Ndc80^ (Figures S1A and S1B available online); however, no increase in lateral kinetochores was observed, and only 10%–15% of kinetochores were laterally attached. This shows that the degree of uncertainty in scoring lateral kinetochores with the monopolar assay is likely to be less than 11% (Figure S1B; codepletion of Mis12 and HEC1^Ndc80^ yielding the highest number of detached kinetochores). We conclude that our fixed-cell assay is sensitive enough to distinguish lateral kinetochores from detached kinetochores that may nonspecifically overlap with microtubules.

To study the biochemical composition of lateral kinetochores, we immunostained cells using CREST antisera and antibodies against β-tubulin and either Mad2 (checkpoint protein lost from kinetochores after attachment) [21] or astrin (plus-end-bound protein associated with attached chromosomes) [14, 22, 23]. In monopolar spindles of both monastrol-treated and untreated cells, Mad2 was present on ∼84% of lateral but not on end-on kinetochores (Figures 1B and 1D; data not shown). This is consistent with Mad2 enrichment selectively on the leading kinetochore [1, 2, 24] that interacts with microtubule walls [3, 4, 16]. Astrin was absent in over two-thirds of lateral kinetochores and present on all end-on kinetochores (Figures 1C and 1D; data not shown). Low levels of astrin found in a small minority of lateral kinetochores (Figure S1C) are consistent with previous observations of mixed lateral attachment wherein a kinetochore engages with both walls and ends of microtubules [4, 25]. Thus, fixed-cell studies of outer-kinetochore composition and kinetochore-microtubule configuration show that monopolar spindles of both monastrol-treated and untreated cells display lateral kinetochores that are similar in their astrin and Mad2 recruitment status.

To assess the extent to which lateral kinetochores are converted to end-on ones in monopolar spindles, we turned to live-cell imaging of HeLa cells expressing YFP-tubulin (microtubule marker) and CENP-B-DsRed (kinetochore marker) (HeLa^YFP-Tub;Cen-Red^), acquired image Z stacks once every 10 s, and tracked three-dimensional (3D) positions of lateral kinetochore and the kinetochore-associated microtubule bundle’s plus end (Figure 1E). While tracking lateral kinetochores, we excluded nonkinetochore microtubules that drifted in and out of the plane of focus. Rarely, change in interkinetochore axis (tumble, T) was observed (Figure 1E). To distinguish end-on and lateral kinetochores in live-cell movies, we asked whether the tubulin signal in the vicinity of the kinetochore ended at the kinetochore (end-on KT) or continued beyond the kinetochore (lateral KT) using 3D rotations and traces of tubulin signal (Figures 1E and 1F). In lateral kinetochores of monastrol-treated cells, the distance between the kinetochore attachment site and the kinetochore-associated microtubule bundle’s plus end (KT-MT end; Figure 1G) was a maximum of 4.2 μm and an average of 2.98 ± 0.5 μm (n_KTs_ = 28; n_cells =_ 17). The ratio of tubulin intensity, along the KT fiber, at positions after and before the site of interaction with the kinetochore showed progressive reduction through time during end-on conversion, indicating a gradual loss of lateral microtubules (Figure 1F). This progressive decrease in lateral microtubule intensity suggests that lateral kinetochores are gradually converted into end-on kinetochores over a period of time.

Time-lapse movies showed that the forward and regressing movements of end-on kinetochores alone were synchronized with the movements of the microtubule end (n_KTs_ = 19; n_cells =_ 13; Figure 1E). We confirmed this synchronous behavior by quantifying the difference in the displacement of kinetochore (ΔKT) and microtubule end (ΔMT) (Figures 1G and 1H). Synchronous movement of the kinetochore and microtubule end was obvious when KT-MT end distance was reduced to <0.2 μm, and maintained at <0.2 μm for at least 30 s (end-on KT); when KT-MT end distance was >0.3 μm (lateral KT), synchronous movement was absent (n_KTs_ = 19; n_cells_ = 13). In summary, we distinguished end-on and lateral kinetochores in our live-cell assay using two criteria (Table S1): First, lateral but not end-on kinetochores showed tubulin signal that extended beyond the centromeric signal. Second, end-on but not lateral kinetochores showed synchronous kinetochore and microtubule-end displacements that lasted for at least 30 s, confirming successful transmission of microtubule length changes into chromosome movement.

The time a lateral kinetochore spends along microtubule walls is variable (n_KTs_ = 45; n_cells_ = 29; Figure 1J). Changes in both kinetochore and microtubule-end positions were observed in lateral kinetochores (Figure 1J). Despite the variability in positions, a lateral kinetochore (L) converted into an end-on kinetochore (E) within 50 s in monopolar spindles of both monastrol treated cells (n_KTs_ = 30/45; n_cells =_ 29) and early mitotic cells without monastrol treatment (n_KTs_ = 21/35; n_cells =_ 10; Figures 1E and 1I). This demonstrates the rapid nature of the end-on conversion process and highlights the similarity of the process in monopolar spindles of monastrol-treated and untreated cells.

We quantified the change in KT-MT end distance and the displacement made by either the microtubule-end (ΔMT) or kinetochore (ΔKT) between consecutive time frames (Figure 1G). Lateral kinetochores displayed variable ΔKT toward the plus and minus end of the microtubule (n_KTs_ = 17/17; n_cells_ = 10) and variable ΔMT associated with microtubule growth and shrinkage (Figure 1J). These observations indicate that the end-on conversion process may critically rely on mechanisms that bring the lateral kinetochore and the microtubule plus end closer to each other (Figure 1K). These findings together with the progressive decrease in lateral microtubule intensity during end-on conversion (Figure 1F) reveal the gradual nature of the end-on conversion process in human kinetochores.

### Lateral Kinetochores Seldom Detach during End-on Conversion

**Figure 2:**
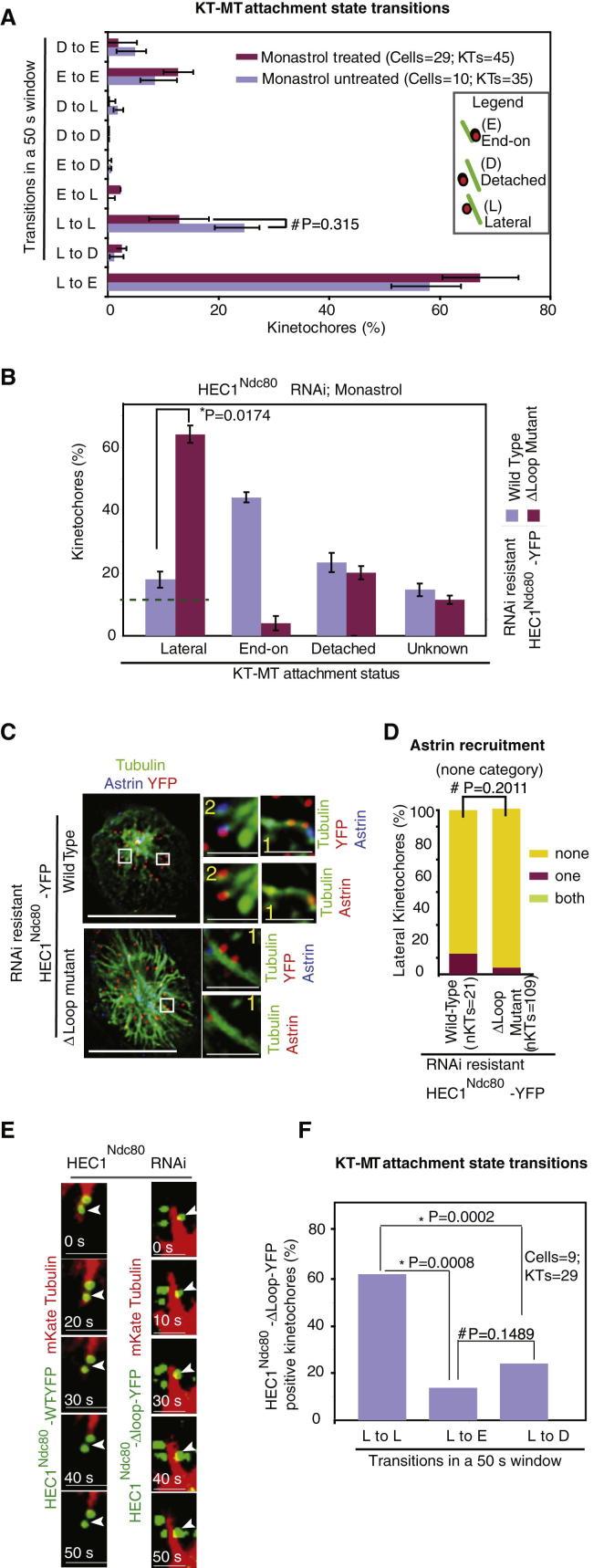
Lateral Kinetochores Seldom Detach during End-on Conversion (A) Graph of transition between attachment states (D, detached; L, lateral; and E, end-on) in lateral kinetochores, within a 50 s window, counted from time-lapse movies of monastrol treated or untreated monopolar spindles. (B) Graph showing the percentage of kinetochores that are detached or attached in a lateral or end-on fashion in monastrol-treated and HEC1^Ndc80^-depleted cells expressing RNAi-resistant YFP fused to HEC1^Ndc80^ -WT or HEC1^Ndc80^-Δloop. Lateral kinetochores were scored using tubulin and HEC1 signals in fixed cells immunostained with antibodies against HEC1^Ndc80^, β-tubulin, and CREST antisera. The green dashed line marks the degree of uncertainty in distinguishing lateral kinetochores in monopolar spindles, despite a large majority of detached kinetochores, as deduced from Figure S1B. (C) Representative immunofluorescence images of cells depleted of endogenous HEC1^Ndc80^ and expressing siRNA-resistant HEC1^Ndc80^-WT-YFP or HEC1^Ndc80^-Δloop-YFP. Cells were treated with monastrol for 1 hr prior to fixation, immunostained with antibodies against GFP (to detect YFP-HEC1^Ndc80^), astrin, and tubulin, and stained with DAPI for DNA. Cropped images are 3× magnified. Insets 1 and 2 are lateral and end-on kinetochores, respectively. Scale bars represent 5 μm (insets, 2 μm). (D) Graph of percentage of lateral kinetochore pairs with both, one, or neither (none) of the pair displaying astrin in cells treated as in (C). (E) Representative images from time-lapse movie of HeLa cells expressing RNAi-resistant HEC1^Ndc80^-WT-YFP or HEC1^Ndc80^-Δloop-YFP (in green) and mKate-tubulin (in red) as indicated, transfected with HEC1^Ndc80^ siRNA, and filmed in the presence of monastrol. White arrowheads mark kinetochores. (F) Graph of percentage of lateral kinetochores that remained lateral (L to L) or converted into end-on (L to E) or detached (L to D), within three consecutive time frames, in movies of HEC1^Ndc80^-depleted cells expressing RNAi-resistant HEC1^Ndc80^-Δloop-YFP. Error bars represent the SEM values across three experimental repeats. p values representing significance level were derived with the Mann-Whitney U test (A) and proportion test for others. ^∗^ and # indicate significant and insignificant differences, respectively.

To define statistically significant events during the end-on conversion process, we counted the transitions a lateral kinetochore made between various microtubule-attachment states, within a 50 s window, in nearly 35 kinetochores (Figure 2A). This population analysis provided a snapshot of key events in the lifetime of a lateral kinetochore, in monopolar spindles of both monastrol-treated and untreated cells: First, end-on attachment was often preceded by a lateral attachment. Second, detachment of lateral kinetochores was less frequent compared to lateral to end-on conversion events (Figure 2A). The population analysis confirmed the similarity of the end-on conversion process in monopolar spindles of monastrol-treated and untreated cells and also highlighted the stable nature of lateral kinetochores during end-on conversion.

End-on conversion study in monastrol-treated cells allowed the enrichment of mitotic cells with monopolar spindles and reduction of whole spindle movements. To further validate our end-on conversion assay in the presence of monastrol, we used mutants that disallowed the formation of end-on attachments. Cells expressing an small interfering RNA (siRNA)-resistant HEC1^Ndc80^ mutant lacking the loop domain (HEC1^Ndc80^-Δloop-YFP) [11, 12] display lateral kinetochores and lack end-on kinetochores. These mutant-expressing cells were depleted of endogenous HEC1^Ndc80^ and exposed to monastrol, and the percentage of lateral attachments was ascertained by immunostaining with antibodies against YFP and β-tubulin and CREST antisera. Analysis of KT-MT attachment status showed a nearly 3-fold increase in lateral kinetochores in HEC1^Ndc80^-Δloop-YFP mutant- compared to HEC1^Ndc80^-WT-YFP (WT, wild-type)-expressing cells (Figure 2B). Next, to investigate whether lateral kinetochores of mutant-expressing cells had collected any attachment to microtubule ends, we analyzed astrin recruitment status using immunostaining with CREST antisera and antibodies against astrin and β-tubulin. Lateral kinetochores of HEC1^Ndc80^-Δloop-YFP-expressing cells lacked astrin (Figures 2C and 2D), confirming the absence of plus-end interaction with kinetochores, consistent with previous reports [11, 12]. Thus, our fixed-cell based end-on conversion assay, using astrin signals, can reliably distinguish end-on and lateral KT-MT attachments.

Next we validated our live-cell assay by confirming the failure in end-on conversion in HEC1^Ndc80^-depleted cells coexpressing mKate-tubulin (microtubule marker) and HEC1^Ndc80^-Δloop-YFP mutant (kinetochore marker). First, in mutant-expressing cells, two to three lateral kinetochore pairs were bound to a common microtubule fiber. Second, the lateral kinetochore and the interacting microtubule end remained distal to each other throughout the filming period of 5 min (n_KTs_ = 29; n_cells_ = 9; Figure 2E). In contrast, end-on conversion was completed within 50 s in HEC1^Ndc80^-depleted cells coexpressing mKate-tubulin and HEC1^Ndc80^-WT-YFP (Figure 2E). In summary, using a previously reported kinetochore mutant that disallows end-on attachments, we show the usefulness of our fixed and live-cell assays in monopolar spindles for studying the end-on conversion process.

Quantification of the transitions in attachment states in HEC1^Ndc80^-depleted cells expressing HEC1^Ndc80^-Δloop mutant showed that lateral kinetochores remained lateral more often than detaching from walls (Figure 2F), revealing the stable nature of kinetochore interaction with microtubule walls. Infrequent detachment of lateral kinetochores during both successful end-on conversion (Figure 2A) and defective end-on conversion (Figure 2F) demonstrates the stable nature of lateral kinetochores. Thus, we conclude that mechanisms that actively mediate end-on conversion should exist in humans because lateral kinetochores remain stably attached and their detachment followed by direct end-on capture is a rare event.

### MCAK and CENP-E Are Required for End-on Conversion

**Figure 3:**
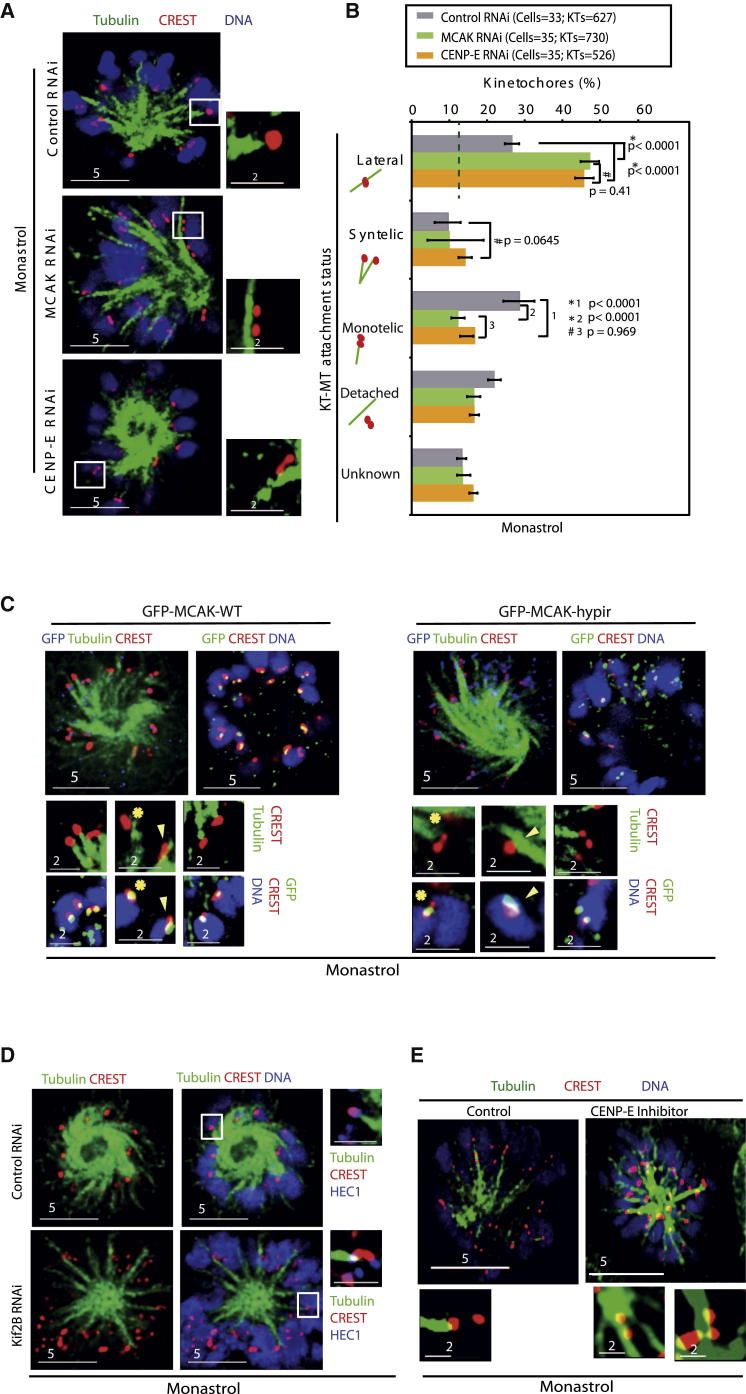
MCAK and CENP-E Activities Are Required for Lateral to End-on Conversion (A and B) Fluorescence images (A) and graph (B) showing the incidence of lateral kinetochores in cells transfected with siRNA oligos as indicated and treated with monastrol alone prior to immunostaining with antibodies against β-tubulin and HEC1^Ndc80^ and CREST antisera and stained with DAPI for DNA. HEC1^Ndc80^ images are not shown in (A) but were used for generating (B). Insets are 3× enlarged. Green dashed line marks the degree of uncertainty in distinguishing lateral kinetochores, as deduced from Figure S1B. (C and D) Fluorescence images showing the incidence of lateral kinetochores in cells transfected with either plasmids encoding MCAK wild-type or dead mutant “hypir” (C) or siRNA oligos (D) as indicated and treated with monastrol for 3 hr, prior to immunostaining with antibodies against β-tubulin, either HEC1^Ndc80^ (D) or GFP (C), and CREST antisera and stained with DAPI for DNA. Insets are 3× enlarged. Yellow arrows in (C) mark lateral kinetochores with MCAK signal in the outer kinetochore along microtubule fiber, and yellow asterisks mark end-on kinetochores with MCAK signal in the inner centromere. (E) Images showing lateral kinetochores in monastrol-treated cells in the presence or absence of CENP-E inhibitors (GSK923295) and immunostained as in (A). Scale bar measurements are indicated. Error bars represent the SEM values across three experimental repeats. p values representing significance level were obtained with the Mann-Whitney U test. ^∗^ and # indicate significant and insignificant differences, respectively. See also Figures S2 and S3.

Kinetochores exhibiting interaction with lateral microtubules were reported in CENP-E- or MCAK-depleted cells [15, 16, 26], but it is not known whether CENP-E or MCAK is required for end-on conversion. We quantified the incidence of lateral kinetochores in cells depleted of either MCAK that depolymerizes microtubules [27, 28] or CENP-E that glides chromosomes toward plus end [16, 29]. Using RNA interference (RNAi) conditions described previously [15, 26, 30–33], we confirmed MCAK and CENP-E RNAi-induced protein depletion of MCAK and CENP-E, respectively, and depletion-induced defective congression in HeLa^YFP-Tub;His-Red^ cells (Figures S2A–S2C). To quantify end-on conversion efficiency in MCAK or CENP-E-depleted cells, we measured steady-state numbers of monotelic, syntelic, lateral, or detached states of KT-MT attachment in monopolar spindles after monastrol treatment. The percentage of laterally attached kinetochores was nearly doubled in cells depleted of MCAK CENP-E compared to control cells (Figures 3A and 3B) or cells depleted of core-kinetochore proteins that detach kinetochores from microtubules (Figure S1B). This shows that the increased incidence of lateral kinetochores after MCAK and CENP-E depletion is not due to an artifactual scoring of detached kinetochores that may nonspecifically overlap with microtubules. We conclude that the abnormally high proportion of lateral kinetochores in CENP-E- and MCAK-depleted cells is indicative of a failure in the end-on conversion process.

### Activities of MCAK and CENP-E Are Important for End-on Conversion

We confirmed MCAK’s specific role in end-on conversion by comparing kinetochore-microtubule attachments in cells expressing GFP fused to either the WT or a mutant of MCAK, GFP-MCAK hypir, bearing three point mutations that inactivate its depolymerizing potential [34]. Immunostaining with anti-GFP antibodies confirmed the localization of MCAK WT and MCAK hypir at outer and inner kinetochores and microtubule plus ends (Figures 3C and S3A). Lateral kinetochores were increased in monastrol-treated cells expressing GFP-MCAK hypir compared to cells expressing GFP-MCAK WT (Figures 3C and S3B), showing that MCAK-mediated microtubule depolymerization is essential for resolving lateral attachments.

To test whether MCAK’s role in end-on conversion is specific and direct, we measured the incidence of lateral kinetochores in cells depleted of Kif2B, a MCAK/Kif2 subfamily member and depolymerizer that destabilizes microtubules in prometaphase and facilitates chromosome congression [35]. Compared to controls, Kif2B-depleted cells did not display an increase in the percentage of lateral kinetochores (Figures 3D and S3C), although Kif2B was depleted and chromosome congression was delayed (Figures S2A and S2B), as reported [35]. We conclude that the microtubule depolymerizer Kif2B is not required for end-on conversion. This study shows that lateral kinetochores in MCAK-depleted cells are not simply an indirect consequence of microtubule stabilization or congression defect, demonstrating a direct role for MCAK in end-on conversion.

To confirm CENP-E’s role in end-on conversion, we compared the percentage of lateral kinetochores found in monastrol-treated cells in the presence or absence of GSK923295, a motor inhibitor [36]. Similar to CENP-E-depleted cells, CENP-E-inhibited cells showed a marked increase in the incidence of lateral kinetochores compared to controls (Figures 3E and S3D), revealing a role for CENP-E motor’s activity in end-on conversion.

Collectively, these findings clearly demonstrate an indispensable role for MCAK and CENP-E activities during end-on conversion in monopolar spindles.

### MCAK and CENP-E Are Required for End-on Conversion in Bipolar Spindles

**Figure 4:**
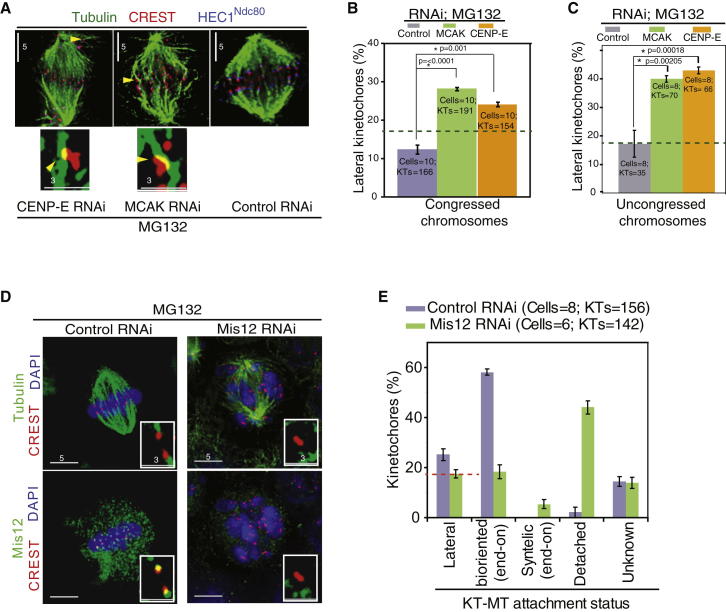
End-on Conversion Failure in Bipolar Spindles of CENP-E- or MCAK-depleted Cells (A–C) Fluorescence images (A) and graphs (B and C) showing lateral kinetochores in congressed (B) and uncongressed (C) chromosomes of bipolar spindles. HeLa cells were transfected with siRNA oligos as indicated, treated with MG132 for 90 min prior to fixation, and then immunostained with antibodies against β-tubulin or HEC1^Ndc80^ and CREST antisera and stained with DAPI for DNA. Scale bar measurements are indicated. Insets are 3× magnified. Yellow triangles mark lateral kinetochores of CENP-E- and MCAK-depleted cells. Error bars represent the SEM values across three experimental repeats. The green dashed line marks the degree of uncertainty in distinguishing lateral kinetochores in bipolar spindles, as deduced from Figure 5E. (D and E) Fluorescence images (D) showing loss of kinetochore-bound Mis12 after Mis12 siRNA treatment and a graph (E) showing lateral, syntelic, bioriented, and detached kinetochores in cells treated with control or Mis12 siRNA. siRNA-treated cells were arrested in metaphase with MG132 for 90 min prior to fixation. Fixed cells were immunostained with antibodies against β-tubulin and Mis12 and CREST antisera and stained with DAPI for DNA. The dashed red line marks the degree of uncertainty in determining lateral kinetochores, in the presence of a large majority of detached kinetochores. Error bars represent the SEM values across two experimental repeats. p values representing significance level were obtained with the Mann-Whitney U test. ^∗^ indicates significant difference.

To test whether the end-on conversion roles of MCAK and CENP-E are restricted to monopolar spindles or are broadly relevant for bipolar spindles as well, we visualized KT-MT attachment status in bipolar spindles. After MG132 treatment, cells were immunostained with antibodies against β-tubulin and HEC1^Ndc80^ and CREST antisera, and the steady-state amount of various KT-MT attachment states in bipolar spindles was quantified. As expected, control-depleted cells predominantly displayed congressed chromosomes, whereas MCAK- or CENP-E-depleted cells displayed both congressed and uncongressed chromosomes (Figures 4A and S2C), as reported [15, 26]. In both uncongressed and congressed chromosomes, lateral kinetochores were more in MCAK- or CENP-E-depleted cells compared to control-depleted cells (Figures 4B and 4C), showing end-on conversion roles for CENP-E and MCAK in bipolar spindles. We confirmed that the percentage of lateral kinetochores in bipolar spindles observed after MCAK or CENP-E depletion was well above the degree of uncertainity in distinguishing a lateral kinetochore (that is physically attached to the microtubule fiber) from a detached kinetochore (that may nonspecifically overlap with tubulin signal). Analysis of bipolar spindles of Mis12-depleted cells treated with MG132 showed congression defects and a 5-fold increase in the percentage of detached kinetochores compared to controls (Figures 4D and 4E). Despite the high incidence of detached kinetochores, less than 15% of kinetochores were laterally attached in Mis12-depleted cells, showing the assay’s sensitivity in distinguishing lateral and detached kinetochores of bipolar spindles. We conclude that lateral kinetochores are more in bipolar spindles of CENP-E- and MCAK-depleted cells compared to control or Mis12-depleted cells. This highlights the role of the two kinesins in end-on conversion in bipolar spindle and further validates the use of monopolar spindles to identify regulators of end-on conversion.

### CENP-E and MCAK Control Distinct Steps during End-on Conversion

**Figure 5:**
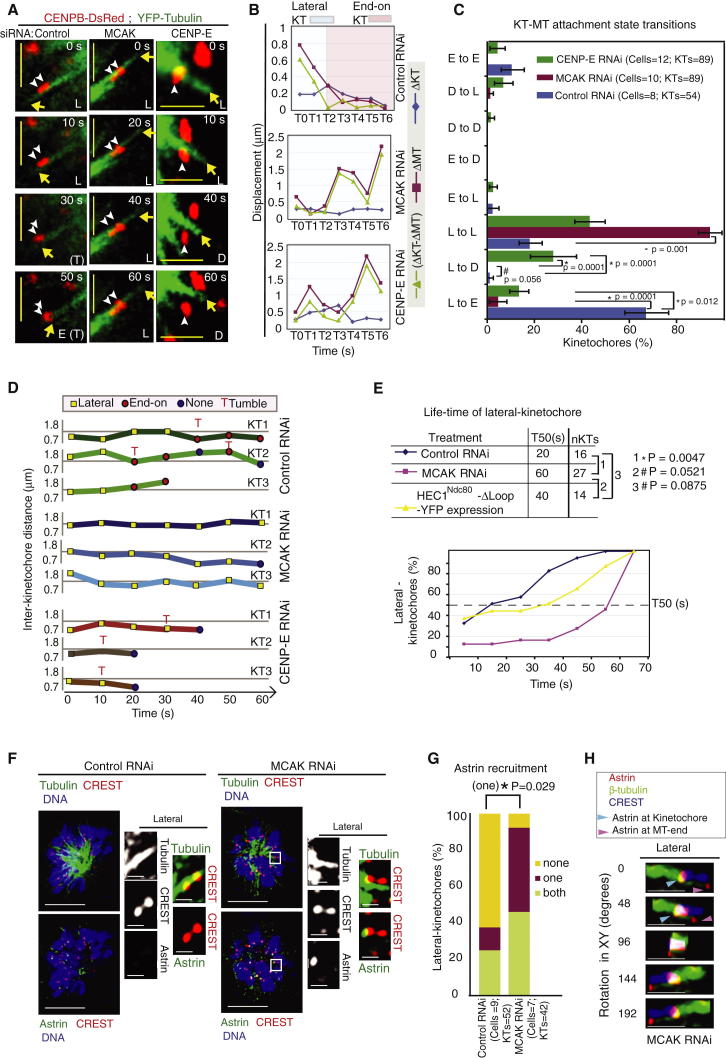
Kinetochores Retain Contact with Lateral Walls of Microtubules in MCAK-Depleted Cells (A) Representative still images of time-lapse movie of HeLa^YFP-Tub;Cen-Red^ cells transfected with siRNA oligos as indicated and filmed in the presence of monastrol. Microtubule tips (yellow arrows) remain distal to kinetochore attachment site in MCAK siRNA-treated cells. Kinetochores (white arrowheads) detach from microtubule walls in CENP-E siRNA treated cells. Lateral, end-on, or no attachment (detached) statuses are marked as L, E, and D respectively. T marks the tumble of kinetochore axis >30°. Scale bars represent 5 μm. (B) Representative graph of ΔKT, ΔMT, and the difference between ΔKT and ΔMT through time in a lateral kinetochore assessed from movie stills as in (A). (C) Graph of attachment state transitions experienced by percentage of lateral kinetochores, within a 50 s window, measured from time-lapse movies of cells transfected with siRNA oligos as indicated and treated with monastrol for 1 hr prior to imaging. (D) Graph of interkinetochore distances describing fate of three randomly chosen kinetochores from conditions described in (A). (E) Cumulative graph and T_50_ table showing lifetime of lateral kinetochores in HeLa cells treated with either control or MCAK siRNA or HeLa cells expressing tetracycline-inducible siRNA-resistant HEC1^Ndc80^-Δloop-YFP as indicated. The dashed line marks T_50_, the period when 50% of lateral kinetochores had ceased to remain laterally attached. (F) Representative immunofluorescence images of control or MCAK siRNA-transfected cells treated with monastrol for 1 hr prior to fixation and immunostained with antibodies against astrin and tubulin and CREST antisera and stained with DAPI for DNA. Insets are 3× magnified. Scale bars represent 5 μm (insets, 2 μm). (G) Graph of the percentage of lateral kinetochore pairs with both, one, or none of the pair displaying astrin in cells treated as in (F). (H) 3D-rendered images are rotated (as indicated) showing a lateral kinetochore in MCAK siRNA-treated cells immunostained with antibodies against astrin (red) and β-tubulin (green) and CREST antisera (blue). The blue arrow marks kinetochore attachment site and pink arrow marks the plus end of laterally interacting microtubules. Scale bars represent 2 μm. p values representing significance levels were obtained with the Mann-Whitney U test (C), proportion test (D and F), and paired sample t test (E). ^∗^ and # indicate significant and insignificant differences, respectively. See also Figure S4.

To determine the precise steps in end-on conversion that are reliant on MCAK and CENP-E, we tracked the fate of lateral kinetochores in MCAK- or CENP-E-depleted HeLa^YFP-Tub;Cen-Red^ cells using time-lapse imaging. In control cells, the lateral kinetochore and its microtubule end were brought proximal to each other within 50 s (n_KTs_ = 24; n_cells_ = 10; Figure 5A). However, in MCAK-depleted cells, the lateral kinetochore and its microtubule end remained distal to each other (n_KTs_ = 24; n_cells_ = 10; Figure 5A). In stark contrast, in CENP-E-depleted cells, lateral kinetochores detached before end-on conversion (n_KTs_ = 24; n_cells_ = 9; Figure 5A). Unlike control-depleted cells, lateral kinetochores in CENP-E- or MCAK-depleted cells showed no synchrony between kinetochore and microtubule-end displacements (Figure 5B), confirming a failure in end-on conversion after the depletion of CENP-E or MCAK.

Analysis of attachment state transitions, within a 50 s window, confirmed that in MCAK-depleted cells alone, the majority of the lateral kinetochores retained lateral attachment (Figure 5C), demonstrating MCAK’s role in removing kinetochore-bound microtubule walls. To assess whether this was simply due to longer or stabilized microtubules in MCAK-depleted cells, we measured microtubule length and dynamics. After MCAK depletion, mitotic cells expressing EB1-YFP (HeLa^EB1-YFP^) displayed an increase in the instantaneous velocity of EB1 comets and the lifetime of EB1 comets (Figures S4A and S4B). This is consistent with MCAK’s role in maintaining catastrophe frequency and with previous vitro observations [37]. As expected, cells treated with monastrol and immunostained with β-tubulin antibody showed that the spindle pole to microtubule-end length was longer after MCAK depletion (upper and lower quartiles were as follows: control RNAi, 3.9–5.1 μm, n_MTs_ = 257, n_cells =_ 22; MCAK RNAi, 4.7–6.2 μm, n_MTs_ = 223, n_cells_ = 21), consistent with previous reports of longer astral microtubules after MCAK depletion [38]. However, time-lapse movies showed that the maximal distance between a lateral kinetochore and its microtubule end per se was not significantly different between control and MCAK-depleted cells (upper and lower quartiles were as follows: control RNAi, 1.5–2.4 μm, n_KTs_ = 28, n_cells =_ 17; MCAK RNAi, 1.9–2.5 μm, n_KTs_ = 31, n_cells_ = 11), ruling out the possibility of long kinetochore fibers interfering with end-on conversion in MCAK-depleted cells. Measurement of interkinetochore distances (indicative of microtubule mediated forces) revealed reduced dynamicity but increased distances in MCAK-depleted cells (1.4 ± 0.3 μm; n_KTs_ = 35; n_cells_ = 19) compared to control-depleted cells (1.1 ± 0.8 μm; n_KTs_ = 35; n_cells_ = 17) (Figure 5D). As previously reported [39], interkinetochore distance measurements distinguish MCAK-depleted cells from cells treated with taxol, a microtubule stabilizer that collapses interkinetochore distances. Thus, we conclude that lateral kinetochores in MCAK-depleted cells maintain dynamic interactions between kinetochore and microtubules and are not an indirect consequence of stable or long microtubules.

### Lateral Kinetochores in MCAK-Depleted Cells Achieve Partial End-on Status

An important insight into MCAK’s function in end-on conversion was revealed from comparison of lateral kinetochores in MCAK-depleted cells and cells expressing HEC1^Ndc80^-Δloop-YFP mutant [11]. While our live-cell assay showed that the lifetime of lateral kinetochores in both MCAK-depleted cells and HEC1^Ndc80^-Δloop-expressing cells is longer than that of control-depleted cells (Figure 5E), analysis of outer-kinetochore composition revealed an important difference. Unlike lateral kinetochores of HEC1^Ndc80^-Δloop-expressing cells that lacked astrin, MCAK-depleted cells displayed astrin on the kinetochore that was facing the pole (compare Figures 2C and 2D with Figures 5F and 5G), indicating the presence of plus ends at the laterally bound kinetochore. Also, when lateral kinetochores of MCAK-depleted cells displayed Mad2 signal, it was only on the kinetochore away from the pole (Figures S4C and S4D). A similar localization pattern was observed for Mad1 as well (Figure S4E). On the basis of Mad2, Mad1, and astrin recruitment status, we conclude that lateral kinetochores in MCAK-depleted cells maintain contact with microtubule walls despite the achievement of a partial end-on status. This is consistent with EM studies of merotelic KT-MT attachments that showed a mixture of lateral and end-on interactions in MCAK-depleted cells [15]. We confirmed the partial end-on status of prometaphase kinetochores in MCAK-depleted cells using 3D-rendered images of astrin-positive kinetochores that retained interaction with microtubule walls. Astrin signal was prominent at the kinetochore attachment site and at the plus-end of the laterally interacting microtubule, revealing the kinetochore’s contact with both ends and walls of microtubules in MCAK-depleted cells (n_KTs_ = 30; n_cells_ = 7; Figure 5H). These findings are consistent with a reduction in tubulin intensity along the kinetochore fiber immediately after the site of kinetochore interaction in MCAK-depleted cells but not HEC1^Ndc80^-Δloop mutant-expressing cells (Figures S4F and S4G). Finally, although MCAK-depleted cells recruit astrin/SKAP to lateral kinetochores, the displacement of the microtubule end and the kinetochore is asynchronous, confirming a failure in translating microtubule changes into kinetochore movement. We conclude that MCAK is required for a late step in end-on conversion to fully remove laterally attached microtubules after a partial end-on attachment is formed.

### Lateral but Not End-on Kinetochores Undergo Detachment in CENP-E-Depleted Cells

**Figure 6:**
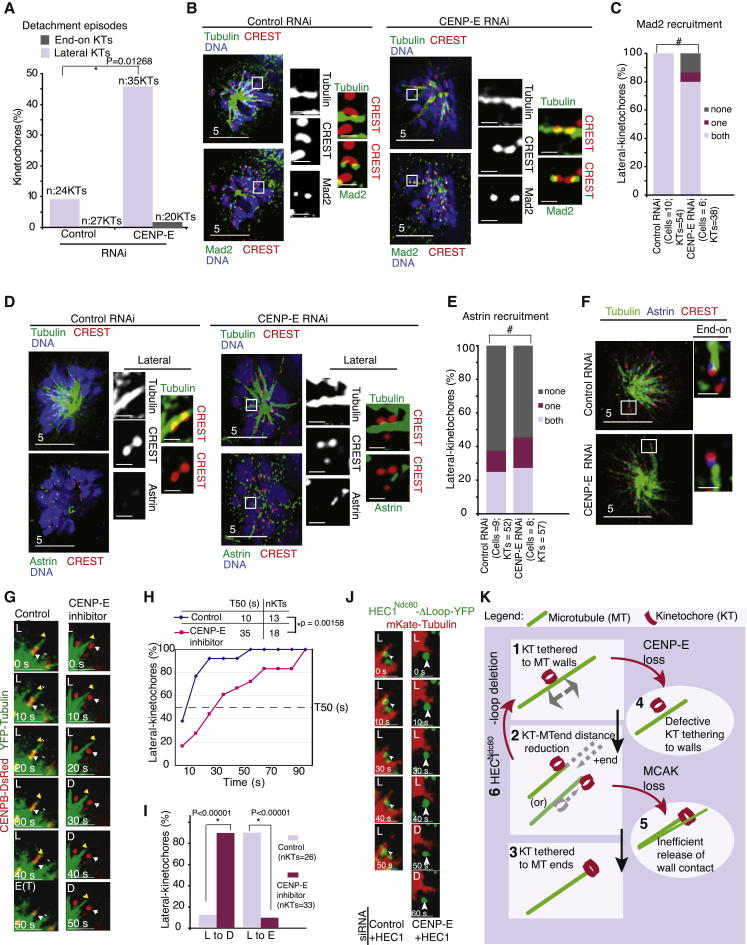
CENP-E Is Required for Tethering Specifically to Lateral Walls of Microtubules (A) Graph of percentage of detachment episodes observed in end-on or lateral kinetochores during the 5 min duration of time-lapse movies of HeLa^YFP-Tub;Cen-Red^ cells transfected with control or CENP-E siRNA. Detachment episodes that lasted at least for three consecutive time frames alone were considered. (B) Immunofluorescence images of control or CENP-E siRNA-transfected cells treated with monastrol for 1 hr prior to fixation and immunostained with antibodies against Mad2 and tubulin and CREST antisera and stained with DAPI for DNA. Cropped images are 3× magnified. Scale bars represent 5 μm (insets, 2 μm). (C) Graph of percentage of lateral kinetochores with both, one, or none of the kinetochores displaying Mad2 in cells treated as in (B). (D and F) Immunofluorescence images of control or CENP-E siRNA-transfected cells treated with monastrol for 1 hr prior to fixation and immunostained with antibodies against astrin and tubulin and CREST antisera and stained with DAPI for DNA. Cropped images are 3× magnified showing lateral (D) and end-on (F) kinetochores. Scale bars represent 5 μm (insets, 2 μm). (E) Graph of percentage of lateral kinetochore pairs with both, one or none of the kinetochores displaying astrin in cells treated as in (D). (G) Representative still images from time-lapse movie of lateral kinetochore in cells treated or untreated with CENP-E inhibitor. Scale bars represent 5 μm. (H and I) Cumulative graph and T_50_ table showing the lifetime of lateral kinetochores (H) and the percentage of lateral kinetochores that detached or converted into end-on (I) in cells treated with monastrol for 1 hr prior to imaging in the presence or absence of the CENP-E inhibitor GSK923295. Detachment episodes that lasted at least for three consecutive time frames alone were considered. (J) Representative still images from a time-lapse movie of HeLa cells expressing siRNA-resistant HEC1^Ndc80^-Δloop-YFP (in green) and mKate-tubulin (in red) as indicated, treated with siRNA as indicated, and filmed in the presence of monastrol. Arrowheads mark lateral kinetochores. Scale bars represent 2 μm. (K) Illustration of a multistep end-on conversion process involving (1) kinetochore tethering to microtubule walls, (2) bringing kinetochore and microtubule plus end proximal to each other for removing wall contact, and (3) kinetochore tethering to dynamic microtubule ends. CENP-E’s role in tethering kinetochores to lateral walls (4) and MCAK’s role in eliminating microtubule walls interacting with kinetochore (5) and HEC1^Ndc80^-loop-dependent tethering of kinetochores to microtubule ends (6) have been assigned sequential steps. Error bars represent the SEM values across three experiments. p values representing significance levels were obtained with the proportion test, except in (H), where the paired sample t test was used. ^∗^ and # indicate significant and insignificant differenced, respectively. See also Figure S5.

In CENP-E-depleted cells, detachment episodes of lateral kinetochores were frequent (Figures 5C and 6A). In agreement, lateral kinetochores of CENP-E-depleted cells were predominantly Mad2 positive, Mad1 positive, and astrin negative (Figures 6B–6F and S5A). Because detachment occurred in lateral but not end-on kinetochores (Figure 6A), we conclude that CENP-E is essential for tethering kinetochores to microtubule walls, but not ends. Although previous studies demonstrated the ability of CENP-E to bind microtubule walls and proposed CENP-E as a linker for both lateral and end-on kinetochore attachments [26, 40], CENP-E’s role in specifically tethering kinetochores to walls, but not ends, has not been demonstrated so far.

Using a CENP-E inhibitor that traps the motor in a microtubule-bound state, we asked whether the wall-tethering defect can be rescued and, if so, whether wall tethering is sufficient for end-on conversion. While inhibitor treatment slightly prolonged the lifetime of lateral kinetochores (Figure 6H), it was insufficient for end-on conversion (L to E) as lateral kinetochores detached before end-on conversion (L to D) (Figures 6G and 6I). This shows a role for CENP-E’s motor activity in the end-on conversion process. Because CENP-E inhibition detached lateral kinetochores, we could not investigate CENP-E mediated transport of lateral kinetochores toward microtubule ends. Nevertheless, these inhibitor studies confirm kinetochore detachment episodes of lateral kinetochores following the loss of CENP-E activity, which has not been reported so far.

We tested whether the wall-tethering defect indicates a novel and direct role for CENP-E in tethering lateral kinetochores. For this purpose, we performed CENP-E depletion in HEC1^Ndc80^-Δloop-mutant-expressing cells that display lateral kinetochores [11, 12] where multiple kinetochores often interact with the same bundle disrupting kinetochore gliding (Figures 2E and S4G). CENP-E depletion alone, but not MCAK or control depletion, caused a dramatic increase in the detachment episodes of lateral kinetochores in HEC1^Ndc80^-Δloop-mutant-expressing cells (Figures 6J, S5B, and S5C). This shows that detachment episodes of lateral kinetochores are specific to CENP-E depletion and also confirms CENP-E’s wall-tethering role as an early event that precedes HEC1^Ndc80^-loop-mediated end-on tethering event. Thus, this study reveals a CENP-E-mediated step in end-on conversion that precedes the step mediated by HEC1^Ndc80^. Also, it distinguishes CENP-E’s role in wall-tethering during end-on conversion from its role in gliding of lateral kinetochores during chromosome congression [16, 41].

In summary, we show evidence for the end-on conversion process to involve at least two previously unrecognized events: first, CENP-E-dependent wall tethering of a lateral kinetochore, and second, MCAK-mediated removal of laterally bound kinetochore fibers.

## Discussion

We developed a methodology to study how a human kinetochore that interacts with microtubule walls becomes subsequently attached to microtubule ends. We first show that laterally attached kinetochores are stable and that they rarely detach. The stable nature of lateral kinetochores emphasizes the need to rethink about how end-on attachments are established. We propose that as a first step in end-on conversion (Figure 6K), lateral kinetochores are kept tethered to microtubule walls by the action of CENP-E, which is enriched on lateral kinetochores ([42] and this study). As a second step, the lateral kinetochore and its associated microtubule end are brought proximal to each other. In this step, MCAK-mediated microtubule destabilization is required to remove laterally attached microtubules. It is possible that MCAK removes curled protofilaments, which could be potential barriers for a kinetochore to change its interaction from the wall to the end. As a third and final step, a kinetochore is kept tethered onto microtubule ends in a HEC1^Ndc80^-loop-dependent manner ([9–12] and this study). In summary, we show that KT-MT attachments are established in a deterministic stepwise process in which CENP-E, MCAK and HEC1^Ndc80^-loop control temporally distinct steps.

We propose the existence of two novel regulatory mechanisms during end-on conversion. First, mechanisms to handover kinetochore tethering from a CENP-E-dependent wall-tethered mode into an Ndc80-loop-dependent end-tethered mode should exist. Second, mechanisms to prevent the premature release of a kinetochore’s contact with walls prior to the formation of an end-on attachment should exist. Thus, kinetochores may employ different strategies to engage with “walls” versus “ends” of microtubules.

Can a monopolar configuration report on end-on conversion in bipolar spindles? Microtubule capture begins in early mitosis when spindles are monopolar and not yet bipolar. So, monopolar spindles are appropriate for studying the end-on conversion process. In support, we find that the kinetics of end-on conversion is similar in lateral kinetochores of early mitotic cells and monastrol-arrested cells. For end-on conversion events that may occur in late stages of mitosis, it is possible that an increased efficiency is provided by kinetochore fiber stabilization through the biorientation process that reduces Aurora-B activity; also, the high density of microtubules near the congression plate may favor direct end-on capture. This is particularly relevant in the case of CENP-E, where we find conspicuous kinetochore detachment in monopolar spindles. We believe that our monopolar-spindle-based end-on conversion assay is relevant to study how end-on attachments form because the same proteins are essential (CENP-E, MCAK, HEC1^Ndc80^) for end-on conversion in monopolar and bipolar spindles.

Around 20–30 microtubules engage with a vertebrate kinetochore [1, 2]. Hence, end conversion is likely to be a gradual process in which some microtubules bound to a kinetochore may remain laterally bound while few others have become end-on (mixed-lateral attachment). Because light microscopy does not permit the visualization of individual microtubules, we developed a live-cell-based behavioral assay and fixed-cell-based astrin recruitment analysis. In the behavioral assay, we harness the information from live-cell “movies” to offset the limitation of “still images” and thereby distinguish a functional end-on attachment from a nonfunctional mixed-lateral attachment. By analyzing the recruitment of the plus-end protein astrin, we show that a pure-lateral attachment (as in HEC1^Ndc80^-Δloop-mutant-expressing cells) could be segregated from a mixed-lateral attachment (as in MCAK-depleted cells). Together, these alternate approaches have allowed a first look into the mechanistic complexity involved in the gradual conversion of human kinetochore-microtubule attachments from a lateral into end-on geometry.

What is the fate of an unresolved lateral kinetochore? Depending on the nature of the kinetochore lesion, the fate of immature lateral kinetochores may considerably vary. For instance, this work and others [11, 12] show that in cells expressing HEC1^Ndc80^-Δloop mutant, unresolved lateral kinetochores severely abrogate the congression of chromosomes. However, in CENP-E-depleted cells, wall tethering alone is perturbed, and so congression is only delayed. In CENP-depleted cells with bipolar spindles, the end-on capture pathway may compensate for the loss of wall-tethering and allow the majority of chromosomes to congress, as reported previously [16]. In contrast, in MCAK-depleted cells, where lateral attachments persist with a partial end-on attachment status, the persistence of the kinetochores’ contact with walls may lead to merotelic attachments; merotely was previously reported in MCAK-depleted cells [15].

Several kinetochore protein depletions are known to promote the incidence of lateral kinetochores in human cells [4, 11–16, 41, 43, 44]. Our live-cell studies shed first insight into three distinguishable steps that when perturbed can promote lateral kinetochores: (1) failure to remove microtubules after a partial end-on attachment is formed, as in MCAK-depleted cells; (2) failure in end-on tethering, such that lateral kinetochores never become end-on tethered, as in HEC1^Ndc80^-Δloop-mutant-expressing cells; and (3) failure to remain wall tethered, and hence kinetochores detach from walls prematurely and are recaptured onto walls as in CENP-E-depleted cells. Future work is required to learn about how the checkpoint recognizes the different steps in end-on conversion.

## Experimental Procedures

### Cell Culture and Synchronization

HeLa cells cultured in Dulbecco’s modified Eagle’s medium (10% fetal calf serum, penicillin, and streptomycin) were plated onto glass-bottomed dishes (LabTek) or 13 mm round coverslips for imaging. For inhibition studies, cells were treated with 10 μM Monastrol (1305, Tocris), 10 μM MG132 (1748, Tocris) or 30 nM GSK923295 (Med Chemexpress). HeLa^YFP-Tub^ and HeLa^YFP-EB1^ were generated by transfection of pEYFP-αtubulin (Clonetech) or VenusYFP-EB1, respectively. HeLa^His2B-GFP; mCherry-Tub^ was generated by transfection of mCherry-αtubulin (Clonetech) into HeLa^His2B-GFP^cells [45] and fluorescence-activated cell sorted. HeLa^YFP-Tub; Cen-Red^ was generated by transient transfection of pDsRed-CenpB (Clonetech) into HeLa^YFP-Tub^. Cells were synchronized with a single aphidicolin (1 μg/ml) block for 24 hr and were then released for 7 hr prior to filming.

### Plasmid and siRNA Transfections

HeLa cells were transfected with siRNAs as described [45, 46]. siRNA oligos used against MCAK (5′-GATCCAACGCAGTAATGGT-3′), CENP-E (5′-AAACACTTACTGCTCTCCAGTTT-3′), HEC1 (5′-GAGTAGAACTAGAATGTGA-3′), and Kif2B (5′-GGCAAGAAGATTGACCTGG-3′) are from Dharmacon, and Mis12 (HSS128129) is from Invitrogen. For control siRNA, negative control oligo (12935-300, Invitrogen) was used. Vectors expressing EGFP-cgMCAK and EGFP-hypir-cgMCAK containing H530A, R534A, and K537A mutations in cgMCAK described in [34] are from Addgene. mKate-tubulin expression vector (Evrogen) was transiently transfected into HeLa cell lines with tetracycline (Tet)-inducible expression of siRNA-resistant HEC1^Ndc80^ wild-type or HEC1^Ndc80^-Δloop mutant. Transiently transfected cells were assessed 24 hr after transfection. Tet-inducible HEC1^Ndc80^ wild-type or HEC1^Ndc80^-Δloop mutant cells were transfected with HEC1 siRNA, transfected with mKate-tubulin expression vector 24 hr later, Tet induced 4 hr later, and imaged 20 hr later.

### Live-Cell Time-Lapse Imaging

Cells were transfected with siRNA oligos or plasmid vectors for 48 or 24 hr, respectively, prior to imaging and were transferred to Leibovitz’s L15 medium (Invitrogen) for imaging at 37°C. For imaging of chromosome and spindle movements, exposures of 0.1 s and at least three Z planes, 3 μm apart, were acquired every 3 min for 4 hr using a 40× NA 0.75 objective on an Applied Precision Deltavision Core microscope equipped with a Cascade2 camera under EM mode. For imaging kinetochore and plus-end dynamics, exposures of 0.04 s and at least ten Z planes, 0.1 μm apart, were acquired every 10 s for 5 min with a 100× NA 1.2 objective on the microscope described above.

### Immunofluorescence and Immunoblotting

For immunofluorescence, antibodies against HEC1^Ndc80^ [20], tubulin (Abcam; ab6160), Mad2 (Bethyl Lab; A300-300A), astrin (Novus NB100-74638), GFP (Roche; 1181446001), SKAP (Atlas HPA042027), and CREST antisera (Europa; FZ90C-CS1058) were used. Images of immunostained cells were acquired with a 100× NA 1.2 objective on a DeltaVision Core microscope equipped with CoolSnap HQ Camera (Photometrics). Volume rendering (*Volocity*) was performed for 3D analysis of KT-MT attachment status. For immunoblotting, antibodies against MCAK [34], tubulin (Sigma; T6557), Kif2B (Abcam AB98214), and CENP-E [20] were used. Immunoblots were developed with fluorescent secondary antibodies (LICOR), and signal intensities were measured with *Odyssey* software (LI-COR). Kinetochore level of astrin was calculated as a ratio with regards to CREST signal intensities, after subtraction of noise with cytoplasmic intensity values, as done in [45].

## Supplemental Information

Document S1. Supplemental Experimental Procedures, Figures S1–S5, and Table S1
