# Lateral to End-on Conversion of Chromosome-Microtubule Attachment Requires Kinesins CENP-E and MCAK

**DOI:** 10.1016/j.cub.2013.11.015

**Published:** 2013-12-02

**Authors:** Roshan L. Shrestha, Viji M. Draviam

(Current Biology *23*, 1514–1526; August 19, 2013)

In this article, the scale bars were missing from the large panels in Figure 1A, the scale bars on the higher-magnification images in Figures 1B and 1C were incorrect, a control siRNA cell was shown in Figure 1B rather than a nontreated cell, and a control image was shown in Figure 1C that also appeared in Figure 5F. The corrected Figure 1 and its legend are included here. None of these errors affects the results or conclusions of the article.

Also, some confusion has arisen over our use of higher-magnification images in our figures. Please note that these illustrate kinetochore-microtubule attachments under the specific conditions of the experiment and can be from different z planes, or from z planes in other cells in the same experiment. Also please note that the white boxes in the larger panels show the same x-y region as in the magnified images in all figures except Figure 2C, where they illustrate areas of end-on (wild-type) or lateral (loop mutant) attachments.

The authors apologize for the above errors and any confusion that may have resulted.

## Figures and Tables

**Figure 1:**
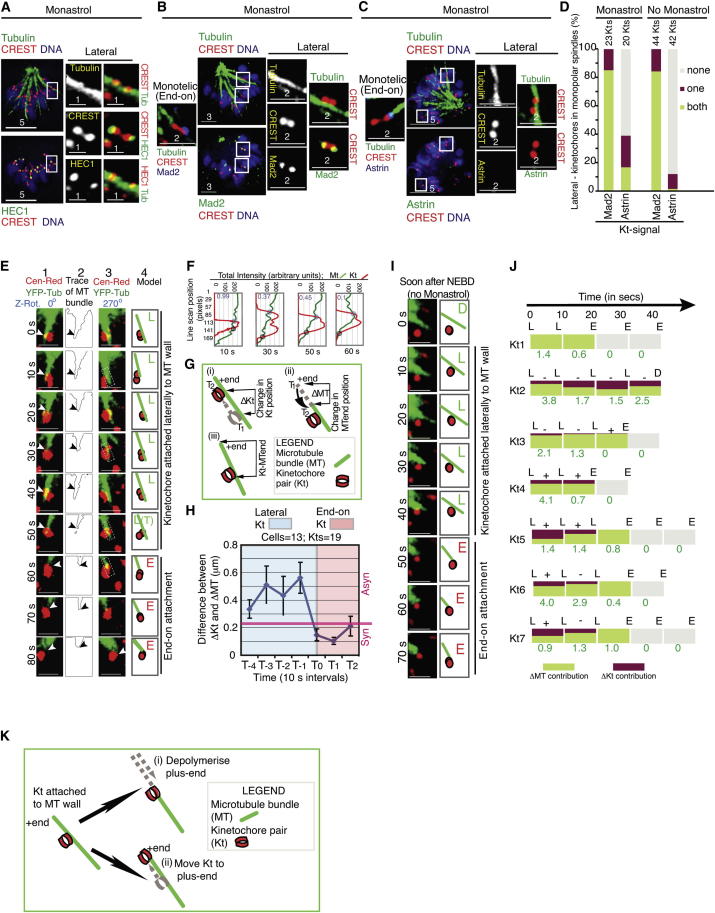
Lateral Kinetochores Are Gradually Converted into End-on Kinetochores (A–C) Fluorescence micrographs showing lateral kinetochores on microtubule walls (A) and levels of Mad2 (B) and astrin (C) on lateral kinetochores in monastrol-treated HeLa cells immunostained with antibodies as indicated and stained with DAPI for DNA. Scale bar measurements are in μm as indicated. Insets show 3× magnifications. (D) Graph of percentage of lateral kinetochore pairs with both, one, or neither (none) of the pair displaying Mad2 or astrin in monopolar spindles of monastrol-treated or untreated cells as indicated. (E) Deconvolved single-plane images of a z stack time-lapse movie of a lateral kinetochore (red) and associated microtubule bundle (green) (column 1) in monastrol-treated HeLa^YFP-Tub; Cen-Red^ cells. Trace of tubulin signal (column 2) illustrates shrinkage and growth of the microtubule bundle. Note the synchronous movement of kinetochore and MT end following end-on attachment (arrows mark kinetochore attachment site). Images in column 3 are 270° z-rotated view of column 1. Models in column 4 mark lateral (L) or end-on (E) kinetochore being tracked. Scale bar represents 2 μm. (F) Graph of total intensity of tubulin and CENP-B signals along the length of rectangular segment (dashed white rectangles at 10, 30, 50, and 60 s time points in column 3 of E). Values in purple indicate ratio of tubulin intensities measured at positions before (blue circle) and after (gray circle) the site of kinetochore interaction with microtubule. (G) Illustration of changes in positions of kinetochore (ΔKt) or microtubule end (ΔMT) between consecutive time frames (T_1_ and T_2_) and distance between the kinetochore attachment site and the microtubule plus end (Kt-MT end). (H) Graph of difference between ΔKt and ΔMT over time during end-on conversion of a lateral kinetochore. T_0_ indicates first time point of end-on attachment. Asynchronous (Asyn) and synchronous (Syn) behaviors indicate greater than and lesser than 0.25 μm, respectively, of ΔKt-ΔMT absolute values. (I) Deconvolved single-plane images of a z stack time-lapse movie of a lateral kinetochore (red) and associated microtubule bundle (green) (left panels) in monopolar spindles of HeLa^YFP-Tub; Cen-Red^ cells soon after nuclear envelope breakdown (NEBD). Models (right panels) mark lateral (L) or end-on (E) kinetochore being tracked. Scale bar represents 2 μm. (J) Temporal evolution of end-on conversion in seven lateral kinetochores selected at random. Fractional contribution of ΔMT (height of green box) and ΔKt (height of red box) to change in Kt-MT end distance in μm (green) is shown. + and − indicate direction of kinetochore movement toward plus and minus end of MT, respectively. (K) Model showing two distinct events, microtubule shrinkage and kinetochore gliding, for bringing the lateral kinetochore and the microtubule end closer to each other. Error bars represent SEM across cells. See also Table S1 and Figure S1.

