# Supplementary material for: Lateral to End-on Conversion of Chromosome-Microtubule Attachment Requires Kinesins CENP-E and MCAK
Source: Curr Biol. 2013 Aug 19;23(16):1514–26. doi: 10.1016/j.cub.2013.06.040 (PMC3748344; doi:10.1016/j.cub.2013.06.040)
Supplement: Document S1. Supplemental Experimental Procedures, Figures S1–S5, and Table S1 [file mmc1.pdf]

Current Biology, Volume *23*

Supplemental Information

Lateral to End-on Conversion of Chromosome-Microtubule Attachment Requires Kinesins CENP-E and MCAK

Roshan L. Shrestha and Viji M. Draviam

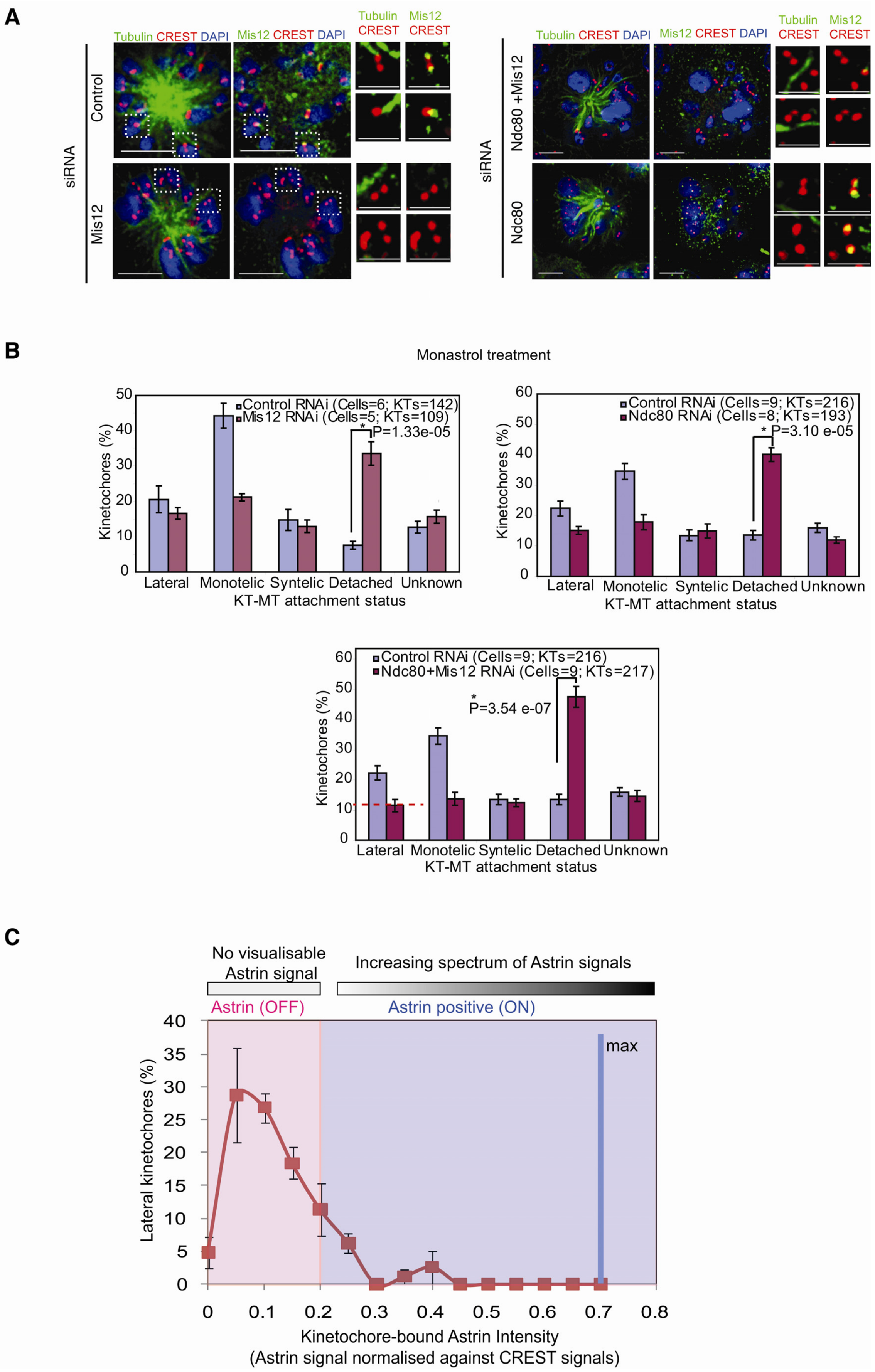
A
Tubulin CREST DAPI
Mis12 CREST DAPI
Tubulin CREST
Mis12 CREST
siRNA
Control
Mis12
Ndc80 +Mis12
Ndc80
B
Monastrol treatment
Control RNAi (Cells=6; KTs=142)
Mis12 RNAi (Cells=5; KTs=109)
* P=1.33e-05
Kinetochores (%)
Lateral Monotelic Syntelic Detached Unknown
KT-MT attachment status
Control RNAi (Cells=9; KTs=216)
Ndc80 RNAi (Cells=8; KTs=193)
* P=3.10 e-05
Control RNAi (Cells=9; KTs=216)
Ndc80+Mis12 RNAi (Cells=9; KTs=217)
* P=3.54 e-07
C
No visualisable Astrin signal
Increasing spectrum of Astrin signals
Astrin (OFF)
Astrin positive (ON)
max
Lateral kinetochores (%)
Kinetochore-bound Astrin Intensity
(Astrin signal normalised against CREST signals)

Figure S1. Lateral-kinetochores are not detached kinetochores that non-specifically overlap with microtubules, Related to Figure 1

A) Images showing levels of kinetochore bound Mis12 in cells treated with siRNA as indicated and exposed to monastrol for 3h before immunostaining. Cells were immunostained with antibodies against Mis12, β-tubulin, CREST antisera and stained with DAPI for DNA. B) Graph of kinetochore-microtubule attachment status showing percentage of laterally, syntelically or monotelically attached or detached kinetochores or kinetochores with unascertainable KT-MT attachment status (unknown) in cells treated as in (A). Scale bar: 5 μm. Error bars represent SEM values across three experiments. Dashed red line marks the degree of uncertainty in determining lateral-kinetochores, in the presence of a large majority of detached kinetochores. p-values representing significance levels were obtained using Proportion test. * and # indicate significant and insignificant difference, respectively. C) Frequency graph shows percentage of lateral-kinetochores with varying intensity of kinetochore-bound Astrin, measured relative to CREST signals. Intensity values were obtained from immunoflourescence images of monastrol treated cells that were fixed and immunostained using antibodies against Astrin, CREST sera and Tubulin. Astrin and CREST intensities show subtracted background noise values before calculating intensity ratio (as described in experimental procedures). Max refers to the maximum possible value, from observations on end-on attached kinetochores. Pink section on left refers to kinetochores where Astrin signals are not visualisable (scored as negative/OFF) and Blue section on right refers to kinetochores were Astrin signals are low yet visualisable (scored as positive/ON). (n: cells = 9; KTs = 56). Error bars represent SEM values across cells.

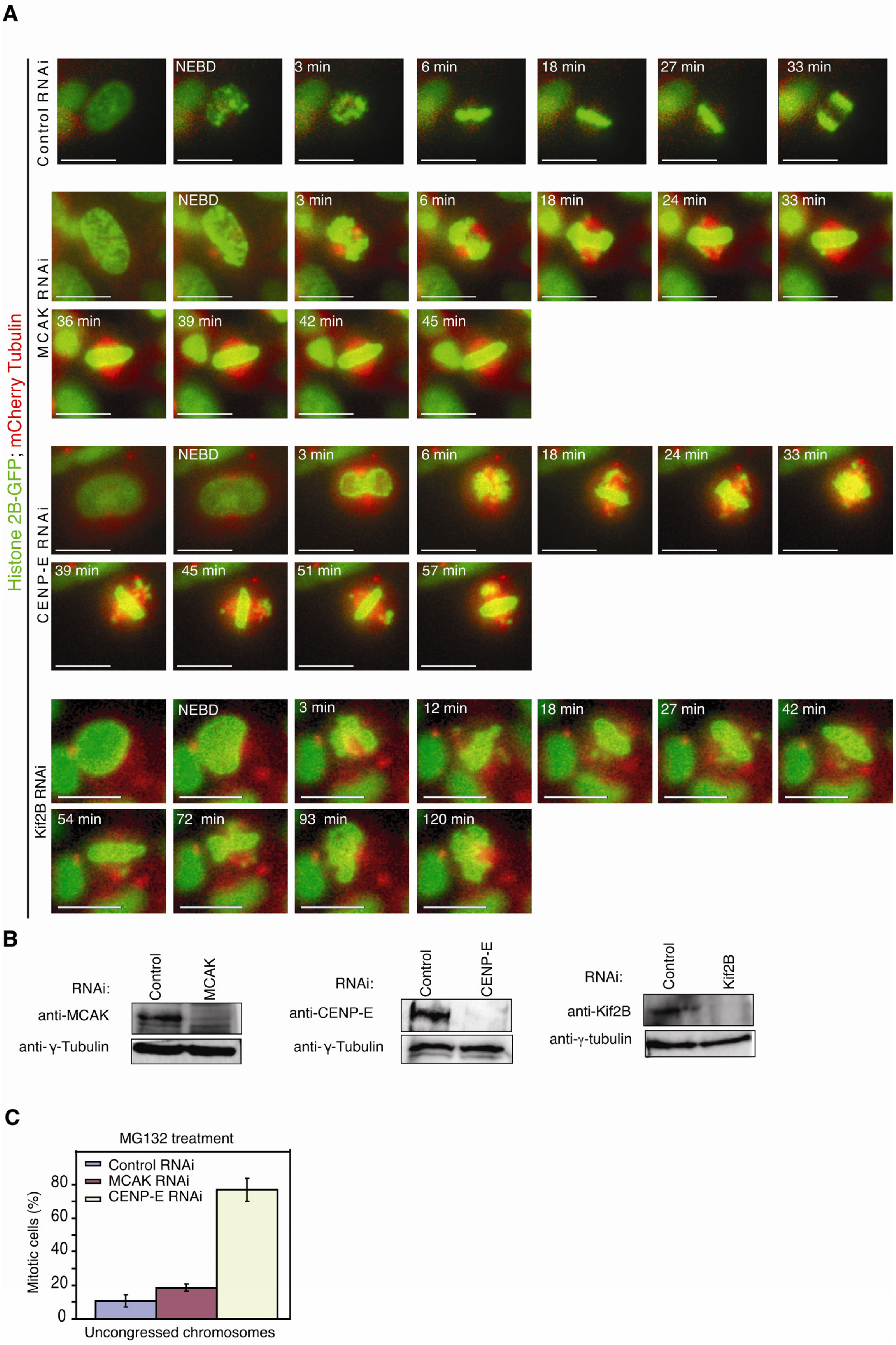
A
Histone 2B-GFP; mCherry Tubulin
Control RNAi
NEBD
3 min
6 min
18 min
27 min
33 min
MCAK RNAi
NEBD
3 min
6 min
18 min
24 min
33 min
36 min
39 min
42 min
45 min
CENP-E RNAi
NEBD
3 min
6 min
18 min
24 min
33 min
39 min
45 min
51 min
57 min
Kif2B RNAi
NEBD
3 min
12 min
18 min
27 min
42 min
54 min
72 min
93 min
120 min
B
RNAi:
Control
MCAK
anti-MCAK
anti-γ-Tubulin
RNAi:
Control
CENP-E
anti-CENP-E
anti-γ-Tubulin
RNAi:
Control
Kif2B
anti-Kif2B
anti-γ-tubulin
C
MG132 treatment
Control RNAi
MCAK RNAi
CENP-E RNAi
Mitotic cells (%)
0
20
40
60
80
Uncongressed chromosomes

Figure S2. MCAK, CENP-E and Kif2B are required for normal congression of chromosomes, Related to Figure 3

A) Representative time-lapse images of HeLa (His2B-GFP; Cherry-Tubulin) cells treated with siRNA as indicated showing congression defects in cells treated with MCAK, CENP-E, or Kif2B as indicated. Scale bar: 10 µm . B) Immunoblots showing extent of protein depletion in lysates of cells treated with siRNA as indicated and probed with antibodies against MCAK, Kif2B or CENP-E, as indicated. Antibody against γ-tubulin was used as a loading control. C) Graph of percentage of mitotic cells displaying congression defects. Cells were siRNA transfected and MG132 treated for 90 min prior to fixing and immunostaining with antibodies against β-tubulin and HEC1^{Ndc80}, CREST anti-sera and DAPI. Congression defect was assigned when one or more chromosomes were unaligned on the metaphase plate. Error bar represents SEM values across three experimental repeats.

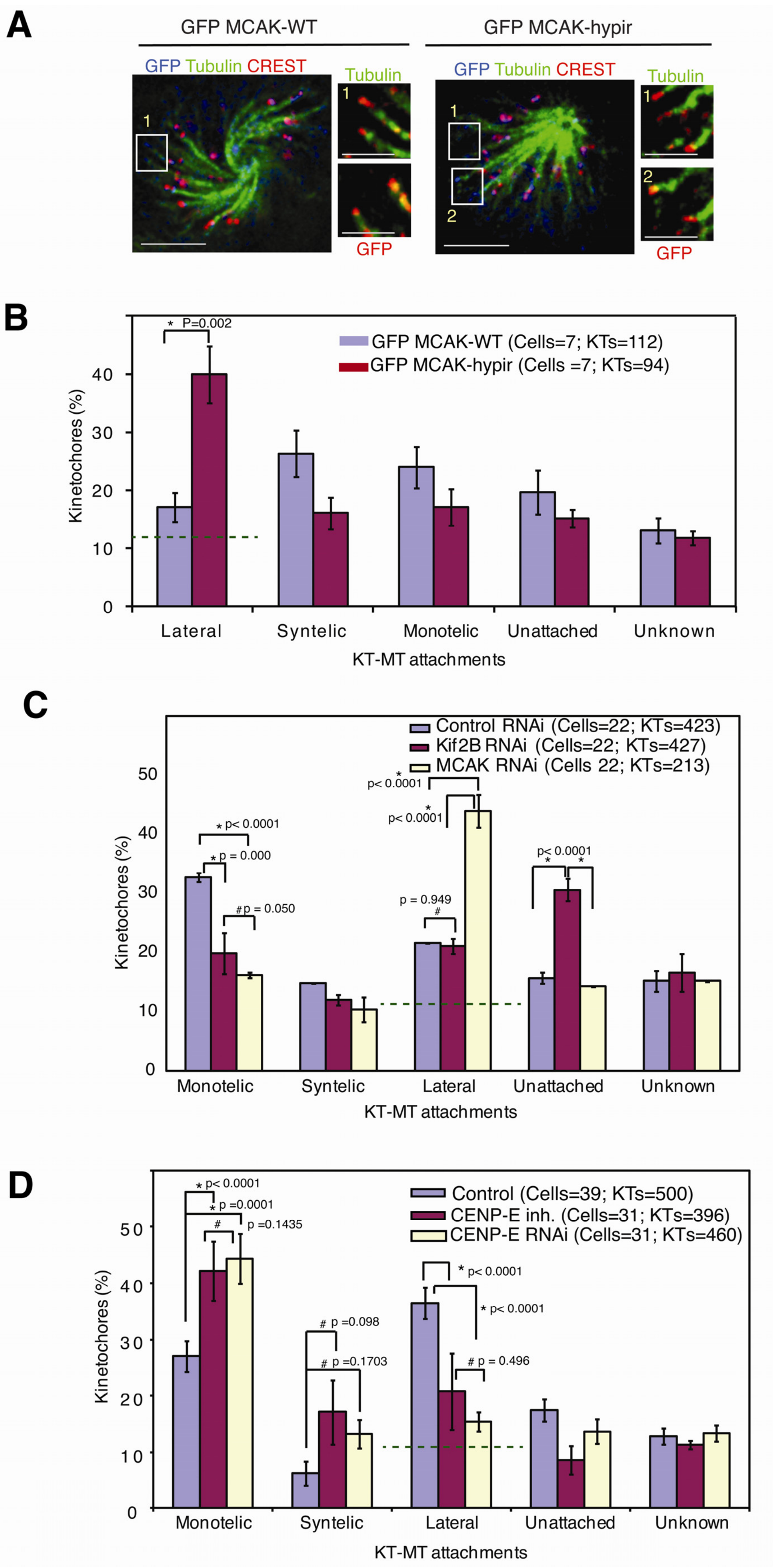
A
GFP MCAK-WT
GFP MCAK-hypir
GFP Tubulin CREST
Tubulin
GFP
B
* P=0.002
GFP MCAK-WT (Cells=7; KTs=112)
GFP MCAK-hypir (Cells =7; KTs=94)
Kinetochores (%)
Lateral
Syntelic
Monotelic
Unattached
Unknown
KT-MT attachments
C
Control RNAi (Cells=22; KTs=423)
Kif2B RNAi (Cells=22; KTs=427)
MCAK RNAi (Cells 22; KTs=213)
* p< 0.0001
* p = 0.000
# p = 0.050
p = 0.949
p< 0.0001
Kinetochores (%)
Monotelic
Syntelic
Lateral
Unattached
Unknown
KT-MT attachments
D
Control (Cells=39; KTs=500)
CENP-E inh. (Cells=31; KTs=396)
CENP-E RNAi (Cells=31; KTs=460)
* p< 0.0001
* p =0.0001
# p =0.1435
# p =0.098
# p =0.1703
* p< 0.0001
# p = 0.496
Kinetochores (%)
Monotelic
Syntelic
Lateral
Unattached
Unknown
KT-MT attachments

Figure S3. Motor activities of MCAK and CENP-E are required for end-on conversion, Related to Figure 3

A) Images showing plus-end localization of MCAK wild-type (WT) and MCAK hypir mutant in cells transfected with plasmids encoding GFP fused to MCAK wild-type or dead mutant 'hypir' as indicated and treated with monastrol prior to immunostaining with antibodies against β-tubulin and GFP (to detect MCAK-GFP), CREST antisera and stained with DAPI for DNA. B-D) Graphs of kinetochore-microtubule attachment status showing percentage of laterally, syntelically, monotelically attached or unattached kinetochores or kinetochores with unascertainable KT-MT attachment status in monastrol-treated cells expressing GFP-tagged MCAK wild-type (WT) or dead mutant 'hypir' (B) immunostained as in Figures S4A, cells treated with Kif2B siRNA or Control siRNA (C) immunostained as in Figure 4D or cells treated with monastrol in the presence or absence of CENP-E inhibitor (inh.), GSK923295 for an hour (D) immunostained as in Figure 4E. Dashed green line marks the degree of uncertainty in determining lateral-kinetochores, as deduced for monopolar spindles in Figure S1. Error bars represent SEM values across three experimental repeats. p-values were obtained using Mann Whitney U test. * and # indicates significant and insignificant difference respectively.

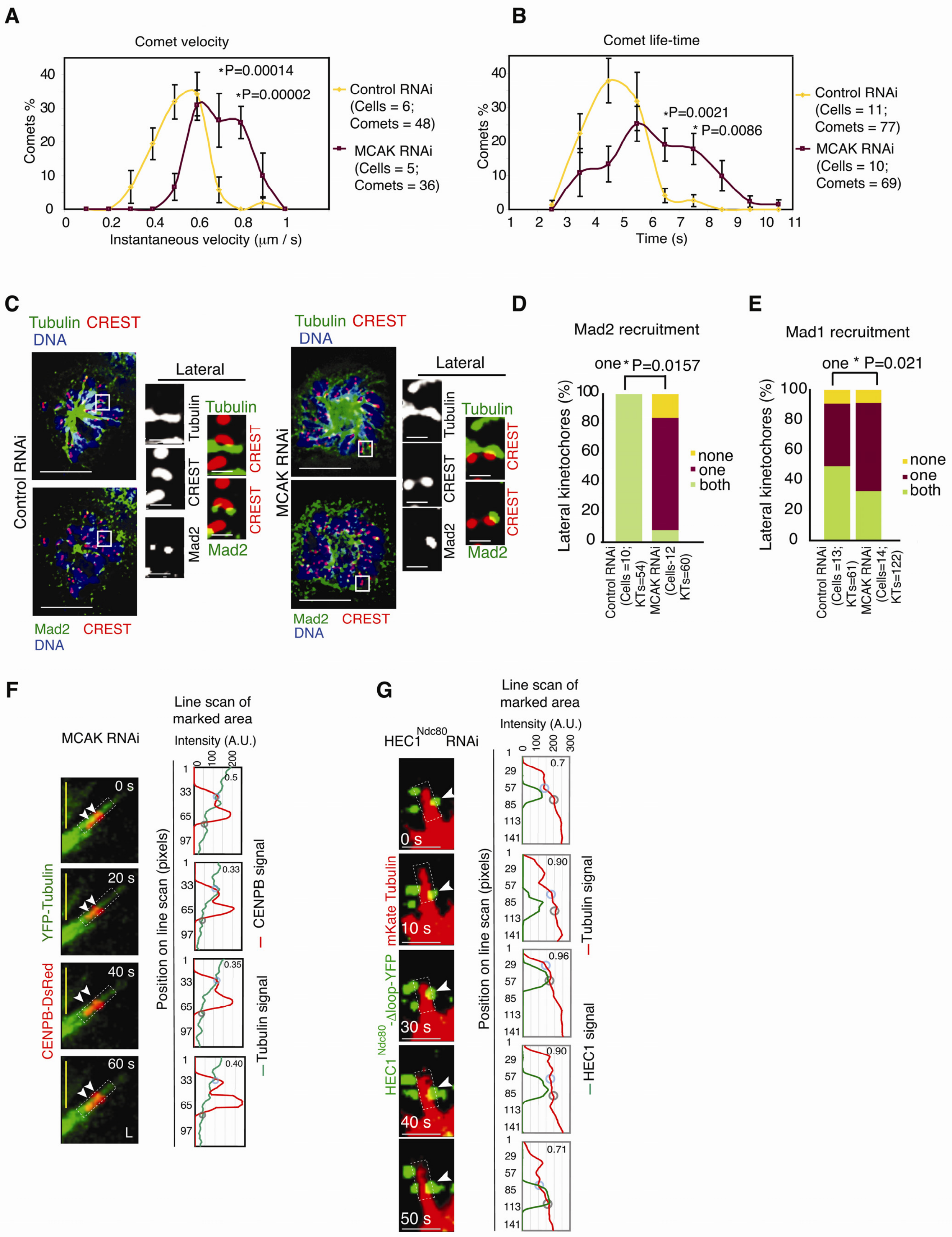

A
Comet velocity
*P=0.00014
*P=0.00002
Control RNAi (Cells = 6; Comets = 48)
MCAK RNAi (Cells = 5; Comets = 36)
Comets %
Instantaneous velocity (μm / s)
B
Comet life-time
*P=0.0021
*P=0.0086
Control RNAi (Cells = 11; Comets = 77)
MCAK RNAi (Cells = 10; Comets = 69)
Comets %
Time (s)
C
Tubulin CREST DNA
Control RNAi
Lateral
Tubulin
CREST
Mad2
Mad2 CREST DNA
MCAK RNAi
D
Mad2 recruitment
one * P=0.0157
Lateral kinetochores (%)
none
one
both
Control RNAi (Cells =10; KTs=54)
MCAK RNAi (Cells-12 KTs=60)
E
Mad1 recruitment
one * P=0.021
Control RNAi (Cells =13; KTs=61)
MCAK RNAi (Cells=14; KTs=122)
F
MCAK RNAi
Line scan of marked area
Intensity (A.U.)
YFP-Tubulin
CENPB-DsRed
0 s
20 s
40 s
60 s
Position on line scan (pixels)
— CENPB signal
— Tubulin signal
G
HEC1Ndc80 RNAi
mKate Tubulin
HEC1Ndc80-Δloop-YFP
0 s
10 s
30 s
40 s
50 s
— Tubulin signal
— HEC1 signal

Figure S4. Lateral-kinetochores in MCAK depleted cells display partial end-on status, Related to Figure 5

A and B) Distribution graph of instantaneous velocity (A) and comet life-time (B) of EB1 comets in $HeLa^{EB1\text{-}YFP}$ cells treated with control or MCAK siRNA as indicated. Cells were treated with monastrol for 3 h prior to filming once every second for a period of 1 min. Error bars represent SEM values across cells. C) Representative immunofluorescence images of Control or MCAK siRNA transfected cells treated with monastrol for 1 h prior to fixation and immunostained with antibodies against Mad2 and Tubulin, CREST antisera and stained with DAPI for DNA. Cropped images are 3x magnified. Bar: 5 µm (in insets: 2 µm). D and E) Graph of percentage of lateral-kinetochore pairs with both, one or neither (none) of the pair displaying Mad2 (D) and Mad1 (E). Cells were treated with siRNA and monastrol as in C and immunostained with antibodies against Tubulin and either Mad2 (D) or Mad1 (E) and CREST antisera. p-values were obtained using Proportion test. * and # indicates significant and insignificant difference respectively. F and G) Image (left panels) of a MCAK depleted $HeLa^{YFP\text{-}Tub;\ Cen\text{-}Red}$ cell (F) and a $HEC1^{Ndc80}$-depleted cell expressing Tet-inducible siRNA resistant $HEC1^{Ndc80}$-Δloop-YFP and mKate-Tubulin (G) treated as described in Figure 6A and Figure 2E respectively. Graphs (right panels) of total intensity of tubulin and CENP-B signals along the length of the rectangular segment (dashed white) of respective images (left panels). Values in purple indicate ratio of tubulin intensity measured at positions before (blue circle) and after (grey circle) the site of kinetochore interaction with microtubule.

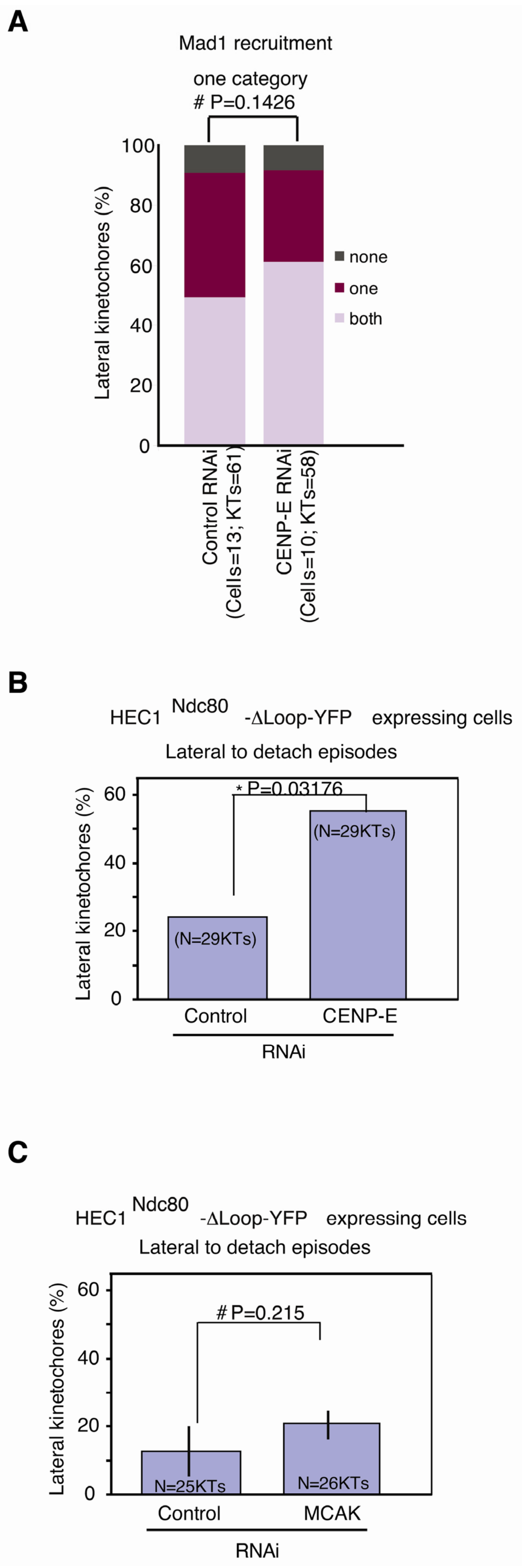
A
Mad1 recruitment
one category
# P=0.1426
Lateral kinetochores (%)
100
80
60
40
20
0
none
one
both
Control RNAi
(Cells=13; KTs=61)
CENP-E RNAi
(Cells=10; KTs=58)
B
HEC1 Ndc80 -ΔLoop-YFP expressing cells
Lateral to detach episodes
* P=0.03176
Lateral kinetochores (%)
60
40
20
0
(N=29KTs)
(N=29KTs)
Control
CENP-E
RNAi
C
HEC1 Ndc80 -ΔLoop-YFP expressing cells
Lateral to detach episodes
# P=0.215
Lateral kinetochores (%)
60
40
20
0
N=25KTs
N=26KTs
Control
MCAK
RNAi

Figure S5. CENP-E is specifically required for wall tethering independent of lateral gliding of kinetochores, Related to Figure 6

A) Graph of percentage of lateral-kinetochore pairs with both, one or neither (none) of the pair displaying Mad1. Cells were treated with siRNA as indicated and treated with monastrol for 3 h prior to fixation. Fixed cells were immunostained with antibodies against β-tubulin and Mad1 and CREST antisera. p-values were obtained using Proportion test. # indicates insignificant difference. B and C) Graph of percentage of detachment episodes observed in lateral-kinetochores during the 5-minute duration of time-lapse movies. HeLa cells co-expressing YFP fused to $HEC1^{Ndc80}$-ΔLoop and mKate-Tubulin were depleted of endogenous $HEC1^{Ndc80}$ and treated with Control or CENP-E siRNA for 36 h (B) or Control or MCAK siRNA for 48 h (C). Detachment episodes that lasted at least for 3 consecutive time-frames alone were considered. Error bars represent SEM values across three experiments repeats. p-values were obtained using Proportion test. * and # indicates significant and insignificant difference, respectively.

Table S1. Criteria for defining lateral, end-on and detached kinetochores, Related to Figure 1

KT-MT attachment states	*Kinetochore vs plus-end movements (live-cells)*	*Geometric configuration of CENP-B and tubulin signals (live-cells)*	*Tubulin intensity before and after site of attachment*	*Signal Overlap between width of HEC1^{Ndc80} and tubulin wall (fixed-cells)*	*Recruitment of plus-end protein, Astrin, to the kinetochore (fixed-cells)*
**1. Lateral**	asynchronous	Tubulin signal extends beyond CENP-B signal.<br>KT-MTend distance: >0.3μ	Similar	Overlap along the entire width of KT present; Tubulin signal extends beyond HEC1^{Ndc80} signal.	Astrin<br>predominantly<br>absent
**2. End-on**	synchronous	Tubulin signal terminates near CENP-B signal.<br>KT-MTend distance: <0.2μ	Dissimilar	Minimal overlap only with a portion of outer kinetochore; Tubulin signal terminates at HEC1^{Ndc80} signal	Astrin<br>always present
**3. Detached**	asynchronous	CENP-B particles move randomly	Irrelevant	Overlap completely absent.	Astrin<br>always absent

Table briefly summarises the five criteria used to distinguish the three different kinetochore-microtubule attachment states as indicated. Conditions used in live- or fixed-cell assays are marked separately.

Supplemental Experimental Procedures

Image analysis: 3D rotation, Deconvolution, volume rendering

Deconvolution of live and fixed-cell images were performed using *SoftWorx.* Z-slices of 0.1 μm apart were acquired. Rotation angles (α, β, γ) of inter-kinetochore axes for measuring i) change in kinetochore axis > 30 (tumble; T) ii) 3D positions of kinetochores for ΔKT and iii) inter-kinetochore distances were obtained using *SoftWorx*. For quantifying ΔKT and KT-MT end distances, measurements were made in 3dimensions along the length of the microtubule fibre. Traces of tubulin signal were obtained using line tracing option in Photoshop or plot profile option in ImageJ. 3D Volume rendering was performed in *SoftWorx.* Pixel intensity of tubulin, CENP-B or HEC1^{Ndc80} for a defined rectangular segment were obtained using *Image J* and total intensity along the length of the segment was computed and plotted using *Excel*.

Statistical Analysis

To confirm that sampling is sufficient, we use two statistical tests: First, for ordinal variables we performed a Mann-Whitney U test to confirm, using p-values, that we have sampled sufficient number of cells for concluding on differences we report. Second, we measure standard error over mean (s.e.m) across experimental repeats and confirm that the differences we report are not only based on differences in mean values, but also the spread of the mean values between experiments. SD values are SEM values obtained across experiments, cells or kinetochores as indicated in legend. p-values representing significance were obtained using Mann-Whitney U test, Proportion test and Paired sample t-test, as appropriate.

PSF measurements

We deduced the lateral (x.y) and axial (z) resolution to be 0.21μm and 0.39μm respectively for Coolsnap camera conditions, and 0.30 μm and 0.43 μm respectively for Cascade2 camera conditions, using 0.1 μm multi-colour beads (TetraSpeck microspheres, Life Technologies) for 405 nm excitation (an average of 3 measurements). For Cascade2 camera, in green (GFP/YFP) channels the lateral and axial resolutions were 0.35 μm and 0.38 μm respectively and in red (mcherry/DsRed) channels the lateral and axial resolutions were 0.32 μm and 0.35 μm respectively.
